# Algometry for the assessment of central sensitisation to pain in fibromyalgia patients: a systematic review

**DOI:** 10.1080/07853890.2022.2075560

**Published:** 2022-05-17

**Authors:** Pablo de la Coba, Casandra I. Montoro, Gustavo A. Reyes del Paso, Carmen M. Galvez-Sánchez

**Affiliations:** Department of Psychology, University of Jaén, Jaén, Spain

**Keywords:** Algometry, central sensitisation, dolorimetry, evoked pain, fibromyalgia

## Abstract

**Introduction:**

The pathophysiology of fibromyalgia (FM) is related to central sensitisation (CS) to pain. Algometry allows assessing CS based on dynamic evoked pain. However, current algometrýs protocols require optimising, unifying and updating.

**Objectives:**

1) identify the dynamic pain measures used most frequently to effectively assess CS processes in FM, and 2) consider the future of the algometry assessing CS in these patients.

**Methods:**

Cochrane Collaboration guidelines and PRISMA statements were followed. The protocol was registered in PROSPERO database (ID: CRD42021270135). The selected articles were evaluated using the Cochrane risk of bias (ROB) assessment tool. The PubMed, Scopus, and Web of Science databases were searched.

**Results:**

Thirty-four studies were selected, including measures such as temporal summation of pain (TSP), aftersensations (AS), spatial summation of pain (SSP), the noxious flexion reflex (NFR) threshold, conditioned pain modulation (CPM), cutaneous silent period (CuSP), and slowly repeated evoked pain (SREP); and evoked pain combined with neuroimaging. Each measure offered various advantages and limitations. According to ROB, 28 studies were of low quality, 3 of moderate quality, and 3 of high quality.

**Conclusions:**

Several pain indicators have been demonstrated to successfully examine CS involvement in FM in the last years. Algometry, especially when it involves diverse body sites and tissues, might provide further insight into (1) the evaluation of psychological factors known to influence pain experience, (2) new dynamic pain indicators, and (3) the simultaneous use of certain neuroimaging techniques. Further research clarifying the mechanisms underlying some of these measures, and homogenisation and optimisation of the algometrýs protocols, are needed.

**KEY MESSAGES**
- Algometry allows for assessing Central Sensitisation by applying dynamic evoked pain.
- The future of algometry could relapse in its combination with neuroimaging.
- Recently-emerged pain indicators should be considered for algometrýs new protocols.

## 1. Introduction

Fibromyalgia (FM) is a syndrome characterised by widespread, persistent and diffuse pain that is usually accompanied by a wide range of physical (e.g. fatigue, insomnia and stiffness) and psychological (e.g. anxiety, depression and cognitive alterations) symptoms [1–3], which are of relevance for its diagnosis [3]. FM seems to be more prevalent in females than males (9:1) [4]. Another hallmark of FM is pain-related hyperresponsivity [5], which underlies two of its characteristic symptoms: (1) allodynia, defined as increased sensitivity to innocuous stimulation; and (2) hyperalgesia or excessive response to painful stimulation, which may be primary or secondary [6]. Primary hyperalgesia refers to sensitisation of nociceptors (peripheral sensitisation), whereas secondary hyperalgesia involves increased responsiveness of the central nervous system (CNS) to painful stimulation (central sensitisation, CS) [7]. While the aetiology of FM remains unknown, its pathophysiology has been widely related to CS processes [8,9]. CS can be defined as an increase in CNS pain responsiveness as a result of altered nociception at the brain and spinal cord levels [7]. Three main CS processes have been distinguished: (1) long-term potentiation of synaptic transmission in the CNS, (2) activation of certain pain facilitatory pathways outside the receptive fields, and (3) dysfunction in descending inhibitory pain mechanisms [10].

Algometry has proven to be a suitable instrument to assess central allodynia and hyperalgesia in FM, as well as in other pain conditions [11,12]. Algometry is often indistinctly referred to as dolorimetry or quantitative sensory testing (QST), with both of those terms also being used in the pain literature [13,14]. While the tender point count (as used for diagnosing FM) is limited only to the evaluation of certain spontaneous pain points *via* the application of pressure [2], algometry evaluates and detects abnormal nociception using various types of pain stimulation in different body locations [15,16], providing a more sensitive, in-depth and exhaustive pain evaluation [17,18]. Besides, pain responses evaluated by algometry have shown strong associations with the clinical pain in FM [19], as well as with improved clinical outcomes after intervention [20].

Algometry examines responses to evoked painful stimulation in order to estimate/quantify the underlying mechanisms involved in different pain conditions. In this way, though algometry has the advantage of offering a reliable and controlled application of pain stimulation [13], it is not always able to discriminate between FM and other similar pain conditions [20,21], being necessary for its combination with tender point count for achieving a high diagnostic accuracy [22].

Several factors have to be considered in algometry, including the type of stimulation (heat, cold, pressure/mechanical, electrical, etc.), algometer surface, application rate, anatomic location of stimulation, and type of tissue to be tested (skin, subcutaneous/deep muscle, bone, or peripheral nerves) [9,23,24]. Furthermore, the moderating role of psychological factors such as pain catastrophizing, fear of pain, anxiety and depression, all of which have a proven influence on pain responsiveness [25,26], should be considered and measured.

Since its inception, algometry has mostly been used as an evoked measure of static pain, evaluating pain response in a basal nociceptive state [27,28]. Threshold and tolerance, defined as the minimal stimulation to induce pain and maximum painful stimulation that can be endured, respectively, are the most common static evoked pain measures [29]. Under different pain modalities, both have been demonstrated to be significantly lower in FM patients than in healthy controls (HC) [18,30,31]. However, neither parameter relates specifically to CS [17], and both are influenced by individual factors such as somatosensory sensitivity (e.g. the higher the mechanosensitivity, the lower the pressure pain threshold), muscle strength, and the peripheral impulse input to the stimulated tissue [24,32,33]. In fact, the evaluation of CS requires an assessment of the increase in pain responsiveness to repeated painful stimulation [8], which may be determined *via* dynamic pain indicators [24,27,28,34,35]. Dynamic evoked pain measures facilitate the assessment of CS processes, such as the increased activity of ascending pain pathways and diminished activity of descending pain mechanisms, as measured by temporal summation of pain (TSP) [35–37], and conditioned pain modulation (CPM) [38,39], respectively. Therefore, the measures of dynamic pain seem suitable to evaluate FM as a CS-related chronic pain condition.

However, although the utility of algometry to examine pathophysiological mechanisms of FM pain is not in dispute, no recent systematic review has examined the algometry techniques used most widely to assess the specific involvement of CS processes in these patients. A systematic review exploring and synthetising the varied data obtained over the last few years on the use of evoked pain measures for assessing CS in FM is needed to optimise, unify and update current QST protocols. Therefore, to determine the potential of algometry as a tool to assess CS, a systematic review of studies published in the last decade using evoked pain measures to assess CS in FM was conducted. The present systematic review aimed to: (1) identify the dynamic evoked pain measures used most frequently to effectively assess CS processes in FM, by reviewing findings published in the past decade, and (2) assess the future potential of algometry as a method to assess CS involvement in these patients.

## 2. Materials and methods

### 2.1. Search strategy

This systematic review was conducted according to the guidelines of the Cochrane Collaboration and reported based on the Preferred Reporting Items for Systematic Reviews and Meta-Analyses (PRISMA) [40]. The inclusion criteria and analyses were specified in advance, and the protocol was registered in the Prospective Register of Systematic Reviews (PROSPERO) international database (registration ID: CRD42021270135). The search string was as follows: central pain sensitisation AND fibromyalgia AND (algometry OR algometer OR dolorimetry OR dolorimeter OR evoked OR experimental OR elicited OR testing OR pressure OR mechanical OR thermal OR heat OR electrical OR cold).

**Figure 1.**
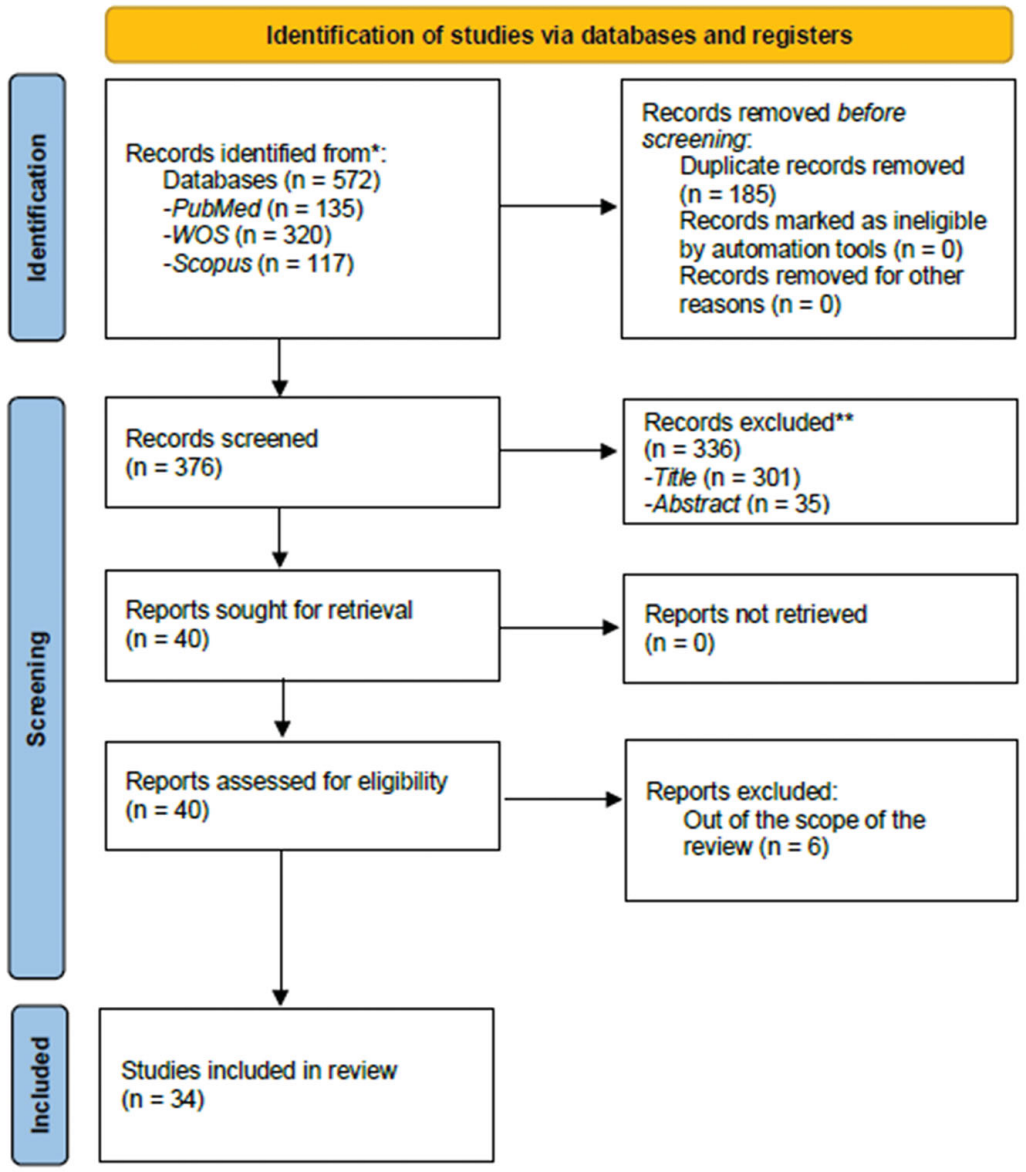
PRISMA 2020 flow diagram for new systematic reviews which included searches of databases, registers and other sources.

The PubMed, Scopus, and Web of Science (WOS) databases were searched independently by two researchers. Discrepancies were resolved by consensus. Two reviewers (P.d.l.C. and C.M.G.S.) independently screened all articles and selected those that satisfied the inclusion criteria for full-text analysis. The titles and abstracts of the articles were screened to remove irrelevant studies (i.e. those clearly unrelated to the aims of this review); the remaining shortlisted articles were screened in depth for eligibility. In order to compile the final set of articles to be reviewed, the full texts of relevant articles were retrieved and screened based on the inclusion and exclusion criteria. Both reviewers decided whether the articles should be included or excluded; any discrepancies were reviewed by the senior author (G.A.R.d.P.), who was the final arbiter regarding the inclusion of a study. The article screening and inclusion processes are illustrated as a PRISMA flowchart (Figure 1). Before data extraction and quality assessment, C.I.M. screened all articles in order to confirm their eligibility for this study. The search was restricted to articles published in the past 2011–2020 decade, and 2021 years. The last search was conducted on December 31, 2021.

### 2.2. Eligibility criteria

Studies were included if they were: (1) peer-reviewed original studies using algometry techniques to assess CS processes in FM patients; (2) full-text publications; (3) published in the period 2011–2021; and (4) published in English. The following studies were excluded: (1) reviews and meta-analyses; (2) comments, editorials, case reports, letters, and meeting abstracts; (3) non-human studies; and (4) non-quantitative studies.

### 2.3. Data extraction and quality assessment

The study characteristics, methodologies and results were extracted independently by P.d.l.C. and C.M.G.S., and any discrepancies were reviewed by G.A.R.d.P. Data were extracted in the following sequence: first author (publication year), country, sample size, age (mean ± SD), sex (%), condition/s, pain modality, body area, device/tool used to evoke pain, details of the procedure, CS measure, primary and secondary outcomes. The study characteristics are shown in Table 1. The data were reviewed by C.I.M. to ensure the accuracy of the extraction thereof.

**Table 1.** Characteristics on the use of dolorimetry to assess central ensitization in fibromyalgia.

First author (year of publication), country. Sample size (*N*), (Age in years old: Mean ± SD), Sex (%). *Condition/s (n).*	❑ Pain modality. ❑ Body area. ❑ Device/tool to evoke pain. Details of the procedure.	❑ CS Measure/s. Primary/secondary outcomes.
Ang et al. (2011), USA [42]. *N* = 32 FM female patients (49.00 ± 11.00). *n = 18 depressed FM patients, n = 14 non-depressed FM patients.*	❑Electrical stimulation. ❑Sural nerve of leg biceps femoris muscle. ❑A constant-current stimulator (DS7A; Digitimer, Welwyn Garden City, United Kingdom). Details: ◆NFR = +1.5 SD in post-stimulation period compared to pre-stimulation one. ◆4-1-mA staircases.	❑NFR threshold. Primary Outcomes: ◆Non-depressed FM patients: the greater clinical pain, the lower NFR thresholds. Secondary Outcomes: ◆Depression attenuates the relationship between clinical pain and NFR.
Blumenstiel et al. (2011), Germany [43]. *N* = 64 female individuals. *n = 21 FM patients (50.60 ± 9.50), n = 23 CBP patients (43.40 ± 8.60), n = 20 HC (38.30 ± 7.60).*	❑Pinprick stimulation. ❑Dorsum of the hand, and back. ❑A stimulator with a flat contact area of 0.25mm-diameter. Details: ◆1 series of 10 pinpricks of 256 mN by site. ◆50% right hand / 50% left hand.	❑TSP/WU. ❑WU ratio. Primary Outcomes: ◆FM and CBP patients: enhanced WU, but not significant WU ratio. Secondary Outcomes: ◆FM patients: ensitizati pain in comparison with CBP patients.
Lambin et al. (2011), Canada [61]. *N* = 100 female individuals. *n = 50 FM patients (44.60 ± 8.30), n = 50 CLBP patients, (43.30 ± 8.10).*	❑Repeated lifting of low-to-moderate weights. ❑General pain increase (most of the body). ❑Canisters of different weight (2.9–3.9-kg). Details: ◆2 lifting tasks of 18 canisters: (a) a pain rating for each lift (with a verbal NRS), (b) an estimate of the weight of each canister. ◆2–5 s of inter-lift intervals. ◆1 min of inter-lifting tasks interval.	❑RISP. Primary Outcomes: ◆FM patients: more RISP than CLBP patients. Secondary Outcomes: ◆RISP was positively correlated with fear of movement and pain disability, but not with depression or catastrophizing.
Sahin et al. (2011), Turkey [65]. *N* = 46 female individuals. *n = 28 FM patients (29.80 ± 7.00), n = 18 HC (30.40 ± 5.10).*	❑Electrical stimulation. ❑Median and ulnar nerves of the abductor pollicis brevis muscle of the right hand. ❑A Nicolet Viking IV current stimulator. Details: ◆Slightly-overstepping of the sensory threshold. ◆5^th^ digital nerve (specific stimulated site). ◆Maintained max. abduction of the thumb.	❑CuSP. Primary Outcomes: ◆FM patients: longer CuSP onset latencies, and delays in the evocation of the inhibitory part of the spinal protective reflex than HC. Non-significant group differences in CuSP duration.
Burgmer et al. (2012), Germany [47]. *N* = 34 female individuals. *n = 17 FM patients (52.59 ± 7.95), n = 17 HC (49.53 ± 8.87)*.	❑Incision followed by mechanical stimulation. ❑Right volar forearm. ❑A Ceramic scalpel (SLG-Ceramic, Bernau) and a 92-mN von Frey mono-filament. Details: ◆A 5–7 mm incision. ◆Mapping along 8 tracks at 45° angles moved towards the incision until the pain perception. ◆A 0–100 NRS.	❑Secondary hyperalgesia + fMRI of pain-related brain areas. Primary Outcomes: ◆FM patients: more secondary hyperalgesia than HC. Secondary Outcomes: ◆FM patients: Secondary hyperalgesia was related to cerebral mid-brain nociceptive mechanisms.
Craggs et al. (2012), USA [52]. *N* = 24 individuals (non-indicated age/sex) *n = 13 FM patients. n = 11 HC.*	❑Heat stimulation. ❑Plantar surface of the right foot. ❑A MR-compatible Peltier thermode with a 30 × 30mm contact surface (TSA-2001; Medoc, Ramat Yishai, Israel). Details: ◆Adjusted intensity to a 45/100 pain. ◆Trains of 6-heat pulses at 0.17–0.33 Hz. ◆A 0–100 NRS.	❑TSP/WU. ❑Activation of pain-related brain areas by fMRI during TSP. Primary Outcomes: ◆FM patients: TSP was not significantly higher than for HC, neither TSP group differences were related to brain activity. Secondary Outcomes: ◆FM patients: TSP was associated with processes involving the left hemisphere and S1, S2, and the posterior insula.
Rhudy et al. (2013), USA [64]. *N* = 55 individuals, 84% women. *n = 18 FM patients (49.44 ± 9.62), 89% women. n = 18 RA patients (44.67 ± 14.32), 83% women. n = 19 HC (46.68 ± 14.14), 79% women.*	❑Electrical stimulation. ❑Sural nerve in the left leg (retromalleolar way). ❑A Constant current stimulator (DS5; Digitimer, Welwyn Garden City, United Kingdom) with a bipolar surface-stimulating electrode (30-mm inter-electrode distance; Nicolet). Details: ◆NFR = response of +1SD in post-stimulation period compared to pre-stimulation one. ◆1–2-mA staircases. ◆Randomised interval inter-stimulations. ◆A NRS.	❑NFR threshold. Primary Outcomes: ◆FM patients: No differences in NFR thresholds regarding RA/HC. Secondary Outcomes: ◆Emotional modulation of the NFR (in all cases). ◆RA patients and HC: Emotional pain modulation (not in FM). ◆FM patients: disrupted supraspinal processing during emotional/pain experiences.
Umeda et al. (2013), USA [71]. *N* = 29 female individuals. *n = 15 FM patients (47.07 ± 12.10), n = 14 HC (41.93 ± 11.46)*.	❑Electrical stimulation. ❑Right biceps femoris muscle. ❑A Constant current stimulator (DS7AH; Digitimer, Welwyn Garden City, United Kingdom). Details: ◆NFR = response of +1SD in post-stimulation period compared to pre-stimulation one. ◆4-2-1-mA staircases.	❑NFR threshold. Primary Outcomes: ◆FM patients: Only 8/15 successfully elicited NFR, while all HC did it. Similar NFR threshold compared to HC. Secondary Outcomes: ◆FM patients: the higher depression, the more difficult to elicit NFR.
Van Oosterwijck et al. (2013), Belgium [72]. *N* = 30 FM patients, 87% women. *n = 15 FM patients receiving physiological education (45.80 ± 9.50), 80% women. n = 15 FM patients receiving pacing self-management education (45.90 ± 11.50), 93% women.*	❑Hot water. ❑Different locations of dominant arm: proximal interphalangeal joints, mid-palm, wrist, distal third of the forearm, middle third of the forearm, elbow, mid-biceps, and shoulder. ❑A system of circulating water. Details: ◆Progressive immersions in 46ºC water. ◆2 min/immersion. ◆A 0–100 NRS (15 s after beginning immersion). ◆5 min of inter-immersion interval.	❑SSP. Primary Outcomes: ◆FM patients: lower SSP who received pain physiology education compared with those who did not receive it.
Desmeules et al. (2014), Switzerland [57]. *N* = 236 individuals, 92% women. *n = 137 FM patients (50.10 ± 9.00), 93% women, n = 99 HC (49.90 ± 10.80), 91% women.*	❑Electrical stimulation. ❑Sural nerve of the retromalleolar tendon. ❑Nicolet Viking IV current stimulator. Details: ◆NFR = >0.5 mV/ms of response. ◆Variable current intensity (from 1 mA). ◆0.5 ms of stimulus duration. ◆6–10 s of inter-stimulus interval. ◆A NRS.	❑NFR threshold. Primary Outcomes: ◆FM patients: lower NFR thresholds than HC. Secondary Outcomes: ◆FM patients: lower NFR thresholds were associated with the Met/Met genotype of COMT Val158Met polymorphism.
Staud et al. (2014), USA [68]. *N* = 71 female individuals. *n = 33 FM patients (42.20 ± 12.60), n = 38 HC (49.10 ± 16.60)*.	❑Repeated heat stimulation. ❑Thenar eminence of both hands. ❑A Medoc (Ramat Yishai, Israel) CHEPS (Contact Heat Evoked Potential Stimulator). Details: ◆6 trains of 5 heat-pulses of 1.5 s at 0.4 Hz. ◆30 s of inter-train interval. ◆A NRS. ◆AS: 15 and 30 s after each train.	❑WU function. ❑AS. Primary Outcomes: ◆FM patients: greater slope of WU function and AS than HC. Secondary Outcomes: ◆FM patients: AS were positively associated with clinical pain.
Coppieters et al. (2015), Belgium [50]. *N* = 59 individuals, 76% women. *n = 21 FM patients (44.52 ± 9.47), 76% women, n = 16 chronic whiplash patients (41.62 ± 11.45), 81% women, n = 22 HC (38.00 ± 13.90), 64% women.*	TSP: ❑Pressure-pulse stimulation. ❑Dorsal side of the intermediate phalanx of the right middle finger, and the middle of the right trapezius belly. ❑A Wagner algometer (Greenwich, CT, USA). CPM: ❑Pressure pulses [T], ischaemic pressure [C]. ❑TSP body locations [T], left arm [C]. ❑The same Wagner algometer [T], an ischaemic occlusion cuff [C]. Details: *TSP:*◆Pulses equivalent to the pain threshold. ◆1 s of inter-pulse interval. ◆A verbal NRS (at 1^st^, 5^th^, and 10^th^ pulses). *CPM:*◆Adjusted cuff inflation to a 3/10 pain.	❑TSP/WU. ❑CPM. Primary Outcomes: ◆FM patients: greater TSP than HC, and the same one that for chronic whiplash patients. ◆Non-significant group differences in CPM.
Üçeyler et al. (2015), Germany [70]. *N* = 70 individuals, 90% women. *n = 25 FM patients (59.00), 92% women, n = 10 MD patients (50.00), 90% women, n = 35 HC (59.00), 89% women.*	❑Pressure stimulation. ❑Muscle bulk of the finger extensors of the dominant hand (right). ❑A Wagner algometer (Greenwich, CT, USA). Details: ◆20 painful/non-painful stimuli of 2 s at 0.1 Hz. (in a ensitizat order). ◆Painful (or not) = 5N above/below the threshold. ◆Different specific location for each series. ◆A 0–100 NRS.	❑O2Hb levels of pain-related brain areas implicated in pain processing to repeated painful pressure stimulation. Primary Outcomes: ❑FM patients: augmented cerebral activation of pain-related brain areas (especially in the dorsolateral prefrontal cortex) in comparison to MD patients and HC, using identical (subjective) painful stimulation.
Bosma et al. (2016), Canada [44]. *N* = 29 female individuals. *n = 14 FM patients (39.00 ± 4.90), n = 15 HC (39.00 ± 10.20)*.	❑Repeated heat stimulation. ❑Thenar eminence of the right hand. ❑An MR-compatible Peltier thermode with a 30 × 30mm contact surface (TSA-2001; Medoc, Ramat Yishai, Israel). Details: ◆Series of 11 heat contacts at 0.33 Hz. ◆Adjusted intensity to a 50/100 pain (42–50ºC). ◆AS: 15 and 30s after each series.	❑TSP/WU. ❑AS. ❑Activation of pain-related brain areas through fMRI during TSP and AS. Primary Outcomes: ◆FM patients: lower intensities to achieve TSP, increased AS, and lower activity in multiple pain inhibition-related brain areas (rostral ventromedial medulla and periaqueductal grey region) and spinal cord (dorsal horn), than HC.
Coppieters et al. (2016), Belgium [51]. *N* = 59 * individuals, 76% women. * The same sample that Coppieters et al. (2015). *n = 21 FM patients (44.52 ± 9.47), 76% women, n = 16 chronic whiplash patients (41.62 ± 11.45), 81% women, n = 22 HC (38.00 ± 13.90), 64% women.*	TSP: ❑Pressure-pulse stimulation. ❑Dorsal side of the intermediate phalanx of the right middle finger, and middle of the right trapezius belly. ❑A Wagner algometer (Greenwich, CT, USA). CPM: ❑Pressure-pulse [T], ischaemic pressure [C]. ❑TSP body locations [T], left arm [C]. ❑The same Wagner algometer [T], an ischaemic occlusion cuff [C]. Details: *See above Coppieters et al. (2015).*	❑TSP/WU. ❑CPM. Primary Outcomes: ◆FM patients: increased TSP in response to relaxation and stressful-cognitive tasks. ◆Chronic whiplash patients and HC: TSP decreased to both tasks. ◆CPM decreased in all groups in response to relaxation and stress. ◆Non-significant group differences in CPM.
Janal et al. (2016), USA [60]. *N* = 174 female individuals. *n = 26 FM + TMD patients (43.40 ± 20.40), n = 99 Myofascial TMD patients (36.30 ± 17.30), n = 48 HC (36.70 ± 14.20)*.	❑Heat-pulses. ❑Thenar eminence of the non-dominant hand, and the skin on the right and left face on the belly of the masseter muscle. ❑A pathway Stimulator (Medoc Ltd., Ramat Yishai, Israel) with a 27mm-diameter thermode. Details: ◆1 °C less than for “late sensations” (or 45ºC). ◆15 heat-pulses trains of 700 ms at 0.5 Hz. ◆A NRS (at 1^st^, 5^th^, 10^th^, 15^th^ stimuli). ◆AS: 15 s after each train.	❑TSP/WU. ❑AS. Primary Outcomes: ◆Similar TSP regardless of FM condition (no group differences). ◆Independent of FM diagnosis: AS decayed more slowly over time in TMD patients than HC.
Montoro et al. (2016), Spain [62]. *N* = 44 female individuals. *n = 24 FM patients (25.31 ± 3.37), n = 20 HC (24.18 ± 3.98)*.	❑Pressure stimulation. ❑Nail of the third finger of the left hand. ❑A JTECH wireless algometer (Midvale, UT, USA) with a 1 cm^2^-surface adapted for manual application of constant increasing pressure. Details: ◆2 series of 12 stimuli of 10 s at 0.017 Hz: (a) using a fixed pressure of 2.4 kg, and (b) using a 6/10 pain (individually calculated).	❑CBF during painful pressure stimuli. Primary Outcomes: ◆FM patients: an anticipatory CBF response in the anterior cerebral arteries, a greater early CBF increase under the fixed pressure condition, a larger CBF decrease after the early component (in both arteries), and a final CBF increase. Secondary Outcomes: ◆FM patients: clinical pain was associated with CBF responses of the medial cerebral arteries.
De la Coba et al. (2017), Spain [53]. *N* = 48 female individuals. *n = 24 FM patients (52.21 ± 9.59), n = 24 HC (50.96 ± 10.27)*.	❑Pressure stimulation. ❑Nail of the 3^rd^ finger of the left hand. ❑A JTECH wireless algometer (Midvale, UT, USA) with a 1 cm^2^-surface adapted for manual application of constant increasing pressure. Details: ◆1 series of 9 weak-moderate painful stimuli of 5 s at 0.03 Hz (30 s of inter-stimulus interval). ◆SREP Pressure = Threshold + 1.25*(DF/4); where DF = Tolerance – Threshold. ◆A 0–10 VAS.	❑SREP ensitization. Primary Outcomes: ◆FM patients: Enhanced pain ensitization to SREP, but not in HC. Secondary Outcomes: ◆SREP ensitization was associated with clinical pain, and had a higher diagnostic accuracy discriminating FM from HC than static measures (controlling pain catastrophizing).
Goubert et al. (2017), Belgium [59]. *N* = 101 individuals, 59% women. *n = 26 FM patients (45.00 ± 9.00), 73% women, n = 23 Recurrent LBP patients (31.00 ± 10.00), 61% women, n = 15 Mild CLBP patients (34.00 ± 10.00), 53 % women, n = 16 Severe Chronic LBP patients (46.00 ± 14.00), 50% women, n = 21 HC (38.00 ± 13.00), 57% women.*	TSP/CPM [T]: ❑Pressure stimulation. ❑Erector spinae, quadriceps, trapezius and hand. ❑A Force Ten algometer (Wagner Instruments, Greenwich, CT, USA) with a 1-cm-circular diameter probe. SSP/CPM [C]: ❑Ischaemic occlusion. ❑Right leg. ❑A computer-controlled cuff algometer (Nocitech and Aalborg University, Denmark) connected to a 13-cm silicone tourniquet cuff (VBM Medizin-technik GmbH, Sulz, Germany), creating a double-chamber cuff.**Details: *TSP:* ◆Series of 10 contacts of pain threshold intensity. ◆A NRS (at 1^st^, 5^th^, and 10^th^ stimuli). *SSP:* ◆Pain threshold/tolerance during inflation of the double-chamber cuff less the values during inflation of the single-chambered cuff. *CPM:* ◆46 °C water. ◆20 s between test and conditioning stimulations. ◆A VAS.	❑TSP/WU. ❑SSP. ❑CPM. Primary Outcomes: ◆FM patients: greater TSP than in CLBP patients and HC. ◆No significant group differences for SSP or CPM.
Schreiber et al. (2017), USA [66]. *N* = 70 individuals, 83% women. *n = 53 FM patients (46.30 ± 11.40), 87% women, n = 17 HC (44.10 ± 14.80), 71% women.*	❑Ischaemic occlusion. ❑Gastrocnemius muscle belly of the left leg. ❑A 13.5-cm-wide Velcro-adjusted blood pressure cuff connected to a cuff inflator (Hokanson Inc., Bellevue, WA, USA). Details: ◆Adjusted cuff inflation to a 40/100 pain. ◆2 min of cuff pressure stimulation. ◆Pain ratings at 60 and 120 s. ◆AS: 15 s after the deflation.	❑TSP. ❑AS. ❑Activation of pain-related brain areas revealed by fMRI during TSP and AS. Primary Outcomes: ◆FM patients: TSP in the majority. Lower pressure to evoke TSP, more pronounced AS, and greater activation of the medial temporal lobe during/after pain stimulation, than HC. Secondary Outcomes: ◆FM patients: AS were positively associated with clinical pain and activation of the medial temporal lobe.
De la Coba et al. (2018b), Spain [54]. *N* = 57 female individuals. *n = 30 FM patients (52.00 ± 9.57), n = 27 HC (51.41 ± 9.94).*	❑Repeated pressure stimulation. ❑Nail of the third finger of the left hand. ❑A JTECH wireless algometer (Midvale, UT, USA) with a 1 cm^2^-surface adapted for manual application of constant increasing pressure. Details: *See above de la Coba et al. (2017).*	❑SREP ensitization. Primary Outcomes: ◆FM patients: Enhanced pain ensitization to SREP, but not in HC. Secondary Outcomes: ◆FM patients: the lower blood pressure, the greater SREP ensitization.
De la Coba et al. (2018c), Spain [55]. *N* = 65 female individuals. *n = 35 FM patients (53.11 ± 8.28), n = 30 RA patients (53.07 ± 10.55).*	TSP: ❑Repeated pinprick stimulation. ❑Thenar eminence of the left hand. ❑A 300-g-calibrated nylon monofilament (Touchtest Sensory Evaluator 6.65). SREP: ❑Repeated pressure stimulation. ❑Nail of the third finger of the left hand. ❑A JTECH wireless algometer (Midvale, UT, USA) with a 1 cm^2^-surface adapted for manual application of constant increasing pressure. Details: *To TSP:*◆2 series of 10 contacts of 1 s at 1 Hz. ◆30 s of inter-series interval. ◆A verbal 0–10 NRS. *To SREP: See above de la Coba et al. (2017).*	❑TSP/WU. ❑SREP ensitization. Primary Outcomes: ◆FM patients: Enhanced pain ensitization to SREP, but not in RA. ◆TSP in both pain conditions. Secondary Outcomes: ◆SREP ensitization had significantly greater test-retest reliability and diagnostic accuracy than TSP to discriminate FM from RA.
Wodehouse et al. (2018), United Kingdom [73]. *N* = *14 FM patients (46.70 ± 10.50), 96% women.*	❑Pressure [T], ischaemic occlusion [C]. ❑The middle part of the right quadriceps femoris muscle [T], left arm [C]. ❑A pressure algometer (type II, Somedic Production AB, Sösdala, Sweden) with a 10-mm diameter tip covered with a 2-mm-thick rubber [T], an ischaemic occlusion cuff [C]. Details: ◆Maintained pain threshold pressure. ◆Ischaemic occlusion at 200 mmHg for 10 min (or until a 6/10 pain).	❑CPM. Primary Outcomes: ◆FM patients: more CPM, and higher pain thresholds and quality of life, after pregabalin treatment.
Brietzke et al. (2019), Brazil [46]. *N* = 69 female individuals. *n = 41 FM patients (49.10 ± 8.32), n = 28 RA patients (32.10 ± 4.56).*	❑Heat [T], maintained cold [C]. ❑Dominant forearm [T], non-dominant hand [C]. ❑An MR-compatible Peltier thermode with a 30 × 30mm contact surface (TSA-2001; Medoc, Ramat Yishai, Israel) [T], cold water [C]. Details: ◆30 s of a 6/10 heat pain. ◆A 0–10 NRS. ◆0–1 °C cold water. ◆1 min of inmersion (30s after heat).	❑CPM. Primary Outcomes: ◆FM patients: lower CPM, and inversely related to BDNF (controlling anxiety, depression, and pain catastrophizing).
Cardinal et al. (2019), Brazil [48]. *N* = 63 female individuals. *n = 17 FM patients (50.50 ± 8.70), n = 18 MD patients (45.20 ± 15.90), n = 28 HC (43.80 ± 13.00)*.	❑Heat [T], maintained cold [C]. ❑Dominant forearm [T], non-dominant hand [C]. ❑An MR-compatible Peltier thermode with a 30 × 30mm contact surface (TSA-2001; Medoc, Ramat Yishai, Israel) [T], cold water [C]. Details: *See above Brietzke et al. (2019).*	❑CPM. Primary Outcomes: ◆FM patients: CPM was significantly lower than in MD patients and HC. Secondary Outcomes: ◆FM patients: higher BDNF than MD. BDNF and CPM were correlated.
Çelik et al. (2020), Turkey [49]. *N* = 49 female individuals. *n =* 21 FM patients (36.80 ± 8.00), *n =* 28 HC (37.30 ± 8.80).	❑Electrical stimulation. ❑Right sural nerve behind the lateral malleolus. ❑An 8-channel electromyograph (Nihon Kohden America Inc., Irvine, CA, USA). Details: ◆Electrical stimulation until 24 times higher than pain threshold, or up to the pain tolerance. ◆5-rectangular-pulses trains of 0.2 ms at 90 Hz. ◆Irregular inter-train intervals (5–30s).	❑NFR threshold. Primary Outcomes: ◆FM patients: lower NFR latency, higher amplitude, wider response area, lower resistance to max. electrical current, and higher pain to max. stimulation, than HC. Secondary Outcomes: ◆FM patients: non-association of clinical symptoms (quality of life, anxiety, and depression) with the NFR threshold.
De la Coba et al. (2020), Spain [56]. *N* = 130* female individuals. * This sample was partially used in de la Coba, 2017, 2018c [53,55*].* *n = 50 FM patients (52.08 ± 8.89), n = 30 RA patients (53.07 ± 10.55), n = 50 HC (50.42 ± 8.12).*	❑Repeated pressure stimulation. ❑Nail of the third finger of the left hand. ❑A JTECH wireless algometer (Midvale, UT, USA) with a 1 cm^2^-surface adapted for manual application of constant increasing pressure. Details:*See above de la Coba et al. (2017).*	❑SREP ensitization. Primary Outcomes: ◆FM patients: Enhanced pain ensitization to SREP, but not in RA/HC. Secondary Outcomes: ◆FM patients: SREP ensitization was positively associated with clinical pain, fatigue, insomnia, and catastrophizing, but not with negative mood. ◆SREP index discriminated FM from RA (controlling clinical pain), and “Fatigue + insomnia + SREP” had a 99% diagnostic accuracy discriminating from FM and HC.
Soldatelli et al. (2020), Brazil [67]. *N* = 117 FM female participants. *n = 60 Non-responders to CPM (48.43 ± 9.29), n = 57 Responders to CPM (50.54 ± 7.84).*	❑Heat [T], maintained cold [C]. ❑Dominant forearm [T], non-dominant hand [C]. ❑An MR-compatible Peltier thermode with a 30 × 30mm contact surface (TSA-2001; Medoc, Ramat Yishai, Israel) [T], cold water [C]. Details: *See above Brietzke et al. (2019).*	❑CPM. Primary Outcomes: ◆Non-responder FM patients (to CPM): discriminated from responders based on a composite index of a set of frequent FM symptoms + another set of neuroplasticity markers with a 100% sensitivity and a 98% specificity (controlling analgesic use, pain threshold, sleep quality, catastrophizing, BDNF levels, FM impact, and psychiatric disorders).
Ydrefors et al. (2020), Sweden [74]. *N* = 50 female individuals. *n = 29 FM patients (38.90 ± 11.70), n = 21 HC (41.20 ± 11.00)*.	❑Electrical stimulation. ❑Foot. ❑A constant current stimulator with a max. output current of 16 mA at a max. compliance voltage of 120 V (Multi Channel Systems MCS GmbH, Reutlingen, Germany). Details: ◆5-square-pulses trains of 1 ms at 200 Hz. ◆2-1-mA staircases. ◆A Z score was derived: NFR = Z score ≥ 12. Z score = difference between the post-stimulation max. amplitude and the pre-stimulation mean amplitude, divided by the SD of the baseline.	❑NFR threshold. Primary Outcomes: ◆FM patients: higher pain, and similar NFR thresholds, than HC. Secondary Outcomes: ◆FM patients: association between NFR thresholds and pain ratings.
Al-Mahdawi et al. (2021), Iraq [41]. *N* = 62 individuals, 73% women. *n = 31 FM patients (age range: 18–62), 74% women, n = 31 HC (age range: 17–55), 71% women.*	❑Constant electrical stimulation. ❑Median nerve of the abductor pollicis brevis muscle of the right hand. ❑An A/S-DK-2740 Keypoint (Medtronic functional Diagnostic, Skovlunde, Denmark). Details: ◆Index finger in constant 50%-contraction. ◆Series of 20 80-mA stimuli of 0.5 ms (1/NFR).	❑CuSP. Primary Outcomes: ◆FM patients: longer CuSP duration than HC. No group differences in CuSP latency. Secondary Outcomes: ◆Sex and age have no significant impact on primary outcomes.
Bourke et al. (2021), United Kingdom [45]. *N* = 70 individuals, 74% women. *n = 19 FM patients (36.00), 84% women, n = 19 chronic fatigue patients (43.00), 68% women, n = 20 HC (34.00), 70% women.*	TSP: ❑Pressure-pulse stimulation. ❑Right quadriceps femoris muscle. ❑17 von Frey monofilaments (0.039–4386 mN). CPM: ❑Pressure [T], ischaemic pressure [C]. ❑Right quadriceps femoris [T], left arm [C]. ❑A pressure algometer with a 10-mm-diameter contact tip, covered with a 2-mm thick rubber [T], an ischaemic occlusion cuff [C]. Details: TSP: ◆A filament to evoke pain. ◆10 contacts of 1 s of duration at 1 Hz. CPM: ◆Cuff occlusion was of 200 mmHg for 10 min (or until a 6/10 pain). ◆A 0–10 NRS (for TSP/CPM).	❑TSP/WU. ❑CPM. Primary Outcomes: ◆FM patients: enhanced TSP and an inefficient CPM was found. No differences in both indicators in relation to chronic fatigue patients. ◆HC: Absence of enhanced TSP or inefficient CPM. Secondary Outcomes: ◆In all: Enhanced TSP was associated with inefficient CPM and vice versa. Non-significant associations between TSP/CPM and current clinical pain, physical function, fatigue or anxiety. ◆Diagnostic properties: CS was detected in 95% of FM patients, 84% of chronic fatigue patients, and 0% of HC.
Donadel et al., (2021), Brazil [58]. *N* = 41 female individuals. *n = 22 FM patients (47.14 ± 9.49), n = 19 HC (34.68 ± 12.45)*.	❑Maintained cold stimulation. ❑Right hand. ❑Cold water. Details: ◆25 °C/5 °C for innocuous/noxious tests. ◆30 s of immersion duration. ◆2 min of inter-test interval. ◆Indices of change in O_2_Hb levels: (1^st^) peak latency; (2^nd^) difference between baseline and max. peak amplitude; and (3^rd^) difference between baseline and 15 s after each stimulation.	❑O_2_Hb levels in pain-related cortical areas implicated in the processing of evoked cold pain. Primary Outcomes: ◆FM patients: diminished cerebral activity at left prefrontal cortex (a pain inhibition-related brain area) than HC. Secondary Outcomes: ◆FM patients: successfully discriminated from HC using the 3^rd^ O_2_Hb index (sensitivity of 85% / specificity of 80%). ◆A 99% diagnostic accuracy to discriminate FM patients with a lower vs. greater severity of CS-related symptoms.
Rehm et al., (2021), Germany [63]. *N* = 87 FM patients (50.40 ± 9.60), 96% women.	❑Repeated pinprick stimulation. ❑Face, hand and foot. ❑A pinprick stimulator with 1 cm^2^ surface area. Details: *Procedure in Rolke et al. (2006).*◆1 train of 10 pinprick stimuli at 1 Hz. ◆128 mN for the face / 256 mN for the hands/feet. ◆A NRS. ◆WU ratio = the pain ratings of the pinprick trains divided by the pain ratings of single stimuli.	❑WU ratio. Primary Outcomes: ◆FM patients with higher and lower clinical pain showed non-significant differences in WU ratio. ◆A 67% ensiti. of FM patients showed CS according to WU ratio.
Staud et al., (2021), USA [69]. *N* = 30 female individuals. *n = 14 FM patients (37.60 ± 16.00), n = 16 HC (48.70 ± 12.80)*.	❑Repeated heat stimulation. ❑Thenar eminence of the right hand. ❑An MR-compatible Peltier thermode with a 30 × 30mm contact surface (TSA-2001; Medoc, Ramat Yishai, Israel). Details: ◆Adjusted intensity to a 50/100 pain. ◆6 trains of 18 stimuli of 1 s at 0.4 Hz. ◆A 0–100 NRS. ◆AS: 30 s after the last stimulus.	❑TSP. ❑AS. ❑Activation of pain-related brain and spinal cord areas by fMRI during TSP. Primary Outcomes: ◆FM patients: lower stimulation to evoke TSP than HC, but not TSP differences. A larger involvement of spinal cord and pain-related brain areas during TSP than HC. AS were presented, but no in HC. ◆No group differences in temporal pattern of spinal activation during TSP. Secondary Outcomes: ◆FM patients: altered descending pain activity in periaqueductal grey matter-rostral ventromedial medulla-dorsal horn.

**Note:** [C] = conditioning stimulation, [T] = Test stimulation. AS = aftersensations, BDNF = brain-derived neurotrophic factor, CBF = cerebral blood flow, CPM = conditioned pain modulation, CS = central ensitization, CUSP = cutaneous silent period, FM = fibromyalgia, fMRI = functional magnetic resonance imaging, HC = healthy control, MD = major depression, NFR = nociceptive flexion reflex, NRS = numeric rating scale, QST = quantitative sensory testing, RA = rheumatoid arthritis, SD = standard deviation, SSP = spatial summation of pain, TMD = temporomandibular disorder, TSP = temporal summation of pain, USA = United States of America, VAS = visual analog scale, WU = wind-up.

In order to evaluate the quality of the selected articles, both C.M.G.S. and C.I.M. independently evaluated the risk of bias (ROB) in each study according to the Cochrane ROB assessment tool. Additionally, P.d.l.C. or G.A.R.d.P. reviewed articles listing C.M.G.S. or C.I.M. as authors. The Cochrane ROB tool contains seven evaluation items: random sequence generation (selection bias), allocation concealment (selection bias), blinding of participants and personnel (performance bias), blinding of outcome assessment (detection bias), incomplete outcome data (attrition bias), selective reporting (reporting bias), and other bias. For each item, the ROB was graded as high, medium or low. Discrepancies were resolved by discussion with the senior author (G.A.R.d.P.), who made the final decision.

### 2.4. Data synthesis

In line with the stated aims of this review, whether the studies used algometry specifically to assess CS to pain in FM patients was analysed. This was done for each individual article identified by the search according to the PRISMA flow diagram (first: title; second: abstract; third: text). The included studies are detailed in Table 1, and evaluated in terms of reporting biases in the Risk of Bias section and Table 2.

**Table 2.** Risk of Bias Assessment of relevant eligible studies.

First author (year)	Random sequence generation (selection bias)	Allocation concealment (selection bias)	Blinding of participants and personnel (performance bias)	Blinding of outcome (detection bias)	Incomplete outcome data (attrition bias)	Selective reporting (reporting bias)	Other bias	General assessment (low, medium, high)
Ang *et al.*, 2011	L	L	H	H	L	L	Y	High
Blumenstiel *et al.*, 2011	H	H	H	H	L	L	Y	Low
Lambin *et al.*, 2011	H	H	H	H	L	L	Y	Low
Sahin *et al.*, 2011	H	H	H	H	L	L	Y	Low
Burgmer *et al.*, 2012	H	H	H	H	L	L	Y	Low
Craggs *et al.*, 2012	H	H	H	H	L	M	Y	Low
Rhudy *et al.*, 2013	H	H	H	H	L	L	Y	Low
Umeda *et al.*, 2013	H	H	H	H	L	L	Y	Low
Van Oosterwijck *et al.*, 2013	L	L	L	L	L	L	Y	High
Desmeules *et al.*, 2014	H	H	H	H	L	L	Y	Low
Staud *et al.*, 2014	H	H	H	H	L	L	Y	Low
Coppieters *et al.*, 2015	H	H	H	H	L	L	Y	Low
Üçeyler *et al.*, 2015	H	H	H	H	L	L	Y	Low
Bosma *et al.*, 2016	H	H	H	H	L	L	Y	Low
Coppieters *et al.*, 2016	L	L	M	L	L	L	Y	High
Janal *et al.*, 2016	H	H	H	H	L	L	Y	Low
Montoro *et al.*, 2016	H	H	H	H	L	L	Y	Low
De la Coba *et al.*, 2017	H	H	H	H	L	L	Y	Low
Goubert *et al.*, 2017	H	H	H	M	L	L	Y	Medium
Schreiber *et al.*, 2017	H	H	H	L	L	L	Y	Low
De la Coba *et al.*, 2018 b	H	H	M	H	L	L	Y	Medium
De la Coba *et al.*, 2018c	H	H	H	H	L	L	Y	Low
Wodehouse *et al.*, 2018	H	H	H	H	L	L	Y	Low
Brietzke *et al.*, 2019.	H	H	M	L	L	L	Y	Medium
Çelik *et al.*, 2020	H	H	H	H	L	L	Y	Low
Cardinal *et al.*, 2019	H	H	H	H	L	L	Y	Low
De la Coba *et al.*, 2020	H	H	H	H	L	L	Y	Low
Soldatelli *et al.*, 2020	H	H	H	H	L	L	Y	Low
Ydrefors *et al.*, 2020	H	H	H	H	L	L	Y	Low
Al-Mahdawi *et al.*, 2021	H	H	H	H	L	L	Y	Low
Bourke *et al.*, 2021	H	H	H	H	L	L	Y	Low
Donadel *et al.*, 2021	H	H	H	H	L	L	Y	Low
Rehm *et al.*, 2021	H	H	H	H	L	L	Y	Low
Staud *et al.*, 2021	H	H	H	H	L	L	Y	Low

*Note.* L = Low, M = Medium, H = High, Y = Yes.

## 3. Results

### 3.1. Literature search and study characteristics

A total of 572 articles were identified by database searches, 376 of which were finally selected for screening after removing duplicates. Figure 1 displays the PRISMA flow chart, which details the number of studies excluded at each stage of the screening process. An analysis of 40 full-text articles was performed to determine their eligibility for the review. Among these articles, 34 fulfilled the inclusion criteria and were subjected to the data extraction (Table 1) and quality assessment processes (Table 2) [41–74]. All of the articles were published between 2011 and 2021, as mentioned above. While 5 studies included only FM patients [42,63,67,72,73], the remaining 29 had one or more control condition/s, such as HC, another chronic pain condition and/or other medical pathologies. Seventeen studies were performed in Europe (Spain, Germany, Belgium, Sweden, Switzerland and the UK), 8 in the USA, 4 in Brazil, 2 in Canada, 2 in Turkey, and 1 in Iraq. All details are provided in Table 1.

### 3.2. Participants

The 34 selected studies on the use of algometry to measure CS to pain in FM included 2177 participants (average of 64 subjects per study; range: 14–236 subjects). Approximately half of the participants were FM patients (*n* = 1134); the others were controls: HC (*n* = 678) and individuals with other chronic medical conditions (*n* = 365). The weighted mean age of all participants was 45.03 ± 7.46 years. In all studies, the age was similar between the patient and control groups, except in 3 studies that reported a significant group difference in age that was not fully controlled for [45,46,58], and 2 studies that did not report participant age [41,52]. Regarding participant sex, 21 studies enrolled only women, 12 other studies had a clear female predominance (59–96%), and 1 study did not report this data [52] (see Table 1).

### 3.3. Algometry in fibromyalgia patients: Evoked pain indicators of Central sensitisation to pain

#### 3.3.1. Temporal summation of pain and indicators derived therefrom

TSP can be defined as an increase in pain perception due to repeated stimulation (> 0.33 Hz), as opposed to the magnitude of these stimuli. The TSP response is based on the wind-up (WU) effect of neurons of the dorsal horn of the spinal cord [75]. It should be noted that TSP usually refers to the temporal summation of second pain, which specifically involves C-fibers [76]. TSP is also referred to as WU [35], and multiple evoked pain indicators are derived from it. Examples include the WU function, which models the WU responses to several series of TSP [68], and the WU ratio, which is based on a comparison between pain ratings in response to a TSP procedure and pain ratings for single stimuli (of the same intensity and in the same body location) [43].

Thirteen articles that used TSP/WU, WU function or the WU ratio as indicators of CS of pain in FM patients were included in the review (see Table 1 for details) [43–45,50–52,55,59,60,63,66,68,69]. Eight of those studies reported a higher TSP/WU, WU ratio or WU function in FMS patients compared to HC [44,45,52,59,66,68] and patients with other medical conditions (chronic low back pain, chronic whiplash, or rheumatoid arthritis) [50,55,59]. However, in three studies, TSP and the WU ratio did not differ between FM, HC, and patients with other medical conditions (chronic fatigue, chronic low back pain, myofascial syndrome, or temporomandibular disorder) [43,45,60,69]. Secondary findings included an increase in TSP after relaxation and cognitive stress tasks in FM patients, a reduction in TSP after these procedures in HC and chronic whiplash patients [51]; and a lack of relationship between TSP and severity of FM symptoms [45], which was contrary to the higher TSP in FM patients reporting more severe clinical pain (although around 33% of these patients did not show a CS response according to the WU ratio) [63].

#### 3.3.2. Repetition-induced summation of activity-related pain

Another TSP-related indicator, which probably also has the WU effect as its underlying mechanism, is the repetition-induced summation of activity-related pain (RISP) [61]. RISP can be defined as a progressive increase in pain ratings over successive weight lifts, even when the overall physical demands of the task remain constant [77].

One article using RISP as an indicator of CS in FM patients was included in our review [61] (Table 1). That study showed that RISP was significantly higher in FM patients than in chronic low back pain patients. Furthermore, RISP was associated with pain disability, but not with depression or catastrophizing in FM patients [61].

#### 3.3.3. Aftersensations (as)

Aftersensations (AS) can be defined as an increase in pain ratings that occurs some seconds after each series of TSP. AS seems to depend on after-discharges of dorsal horn neurons following repetitive C-fiber activation [37].

Five articles that used AS as indicator of CS in FM patients were included in our review [44,60,66,68,69] (Table 1). Among these articles, four reported higher AS in FMS patients compared to HC [44,66,68,69], while and did not show a difference in AS between TMD patients suffering versus not suffering from FM [60]. A secondary finding was that AS were positively associated with clinical pain in FM patients [66,68].

#### 3.3.4. Spatial summation of pain

Spatial summation of pain (SSP) is defined as an increase in pain intensity due to an increase in the size of the stimulated body area, which also depends on the activation of C-fiber in the spinal cord [78,79]. SSP also seems to involve the central mechanism known as diffuse noxious inhibitory control. As well as a progressive increase in the size of the stimulated area, factors like pain intensity and surface size also are relevant to evoking SSP [80].

Two articles that used SSP as an indicator of CS in FM patients were included in our review [59,72] (Table 1); neither article reported significantly higher SSP in FM patients compared to HC and chronic low back patients [59]. However, Van Oosterwijck *et al.* [72] found lower SSP in FM patients who had received an educational intervention on the physiology of pain.

#### 3.3.5. Conditioned pain modulation

CPM is defined as inhibition of the pain produced by a painful (test) stimulus due to the presentation of another (conditioning) stimulus. CPM reflects the level of pain reduction elicited by stimulus interference [81]. Although CPM is mainly used as an inhibitory pain modulation paradigm, its underlying processes involve both complex central facilitatory and inhibitory mechanisms of pain processing, such as diffuse noxious inhibitory control; thus the specific mechanisms involved in this pain measure have not yet been elucidated [81,82].

Eight articles that used CPM as an indicator of CS in FM patients were included in our review [45,46,48,50,51,59,67,73] (Table 1). Among these studies, three reported significantly lower CPM in FMS patients compared to HC and patients with other medical conditions (major depressive disorder or rheumatoid arthritis) [45,46,48]. Two studies did not report CPM differences among FM patients, HC and patients with other medical conditions (chronic low back pain or chronic whiplash) [50,59]. Moreover, a study in which CPM was used as a marker of clinical improvement in FM patients after pregabalin treatment reported a significant recovery in CPM after the treatment [73]. Lower CPM after relaxation and stressful tasks in FM, HC and chronic whiplash patients were observed in another study, but there were no significant differences in CPM among these groups [51]. As secondary findings, CPM was inversely associated with brain-derived neurotrophic factor (BDNF) in three studies [46,48,67], and with some clinical FM symptoms in one study [67]. Additionally, CPM seemed to be moderated by depression levels [48].

#### 3.3.6. Noxious flexion reflex threshold

The noxious flexion reflex (NFR) threshold is defined as the intensity of electrical stimulation required to elicit the NFR. NFR is a polysynaptic spinal withdrawal reflex elicited by the activation of nociceptive A-delta afferents (monitored using electromyography) in response to electrocutaneous stimulation of varying intensity applied to the ipsilateral sural nerve of the biceps femoris muscle. Thus, the NFR threshold indicates the magnitude of the excitatory component of the spinal protective reflex [83]. However, there is no consensus definition of the NFR threshold, varying according to factors such as the electromyographic criteria and the required duration of the NFR response. This makes it difficult to compare this indicator between studies [84].

Six articles using the NFR threshold as an indicator of CS in FM patients were included in our review [42,49,57,64,71,74] (Table 1). Among these studies, two reported a lower NFR threshold in FM patients compared to HC [49,57], while three did not observe NFR threshold differences between FM patients and HC [64,71,74]. At this juncture, it should be noted that Ang *et al.* found that depression had a confounding effect on the inverse relationship between the NFR threshold and FM pain; this association was only observed in non-depressed FM patients [42]. As a secondary finding, a lower NFR threshold was associated with more severe clinical pain [57,71] and pain sensitivity [49], although there were no associations with other clinical FM symptoms [49]. Some studies showed that depression and emotional states modulate these relationships [57,64,71]. Additionally, and in accordance with the influence of depression on the NFR response, FM patients with lower NFR thresholds were more likely to possess the Met/Met genotype of the COMT Val158Met polymorphism (this was less common among individuals with depression) [57].

#### 3.3.7. Cutaneous silent period

The cutaneous silent period (CuSP) can be defined as the brief interruption in voluntary muscle contraction that follows the painful electrical stimulation of a cutaneous nerve, i.e. the inhibitory component of the NFR [85]. The mechanisms underlying CuSP are related to sensorimotor integration processes occurring at the spinal and supraspinal levels [86].

Two articles using CuSP as an indicator of CS in FM patients were included in our review [41,65] (Table 1). Differences in CuSP onset latencies between FM and HC, but not in CuSP duration, were found by Sahin *et al.* [65]. Contrary, Al-Mahdawi *et al.* observed differences in CuSP duration between FM and HC, but not in CuSP onset latencies [41].

#### 3.3.8. Slowly repeated evoked pain sensitisation

Slowly repeated evoked pain (SREP) sensitisation is reflected in an increase in pain ratings in response to repeated evoked pressure stimuli at a frequency of 0.03 Hz. The mechanisms underlying SREP sensitisation are still unknown. This dynamic pain indicator is currently in the latter stages of development. Although a lower stimulation frequency is used in the SREP protocol compared to TSP, WU is not considered a possible underlying mechanism of SREP sensitisation. Long-term potentiation of CS processes has been proposed [54]. SREP sensitisation showed ≥ 85% diagnostic accuracy for discriminating FM patients from HC and patients with other non-CS chronic pain conditions [53,55,56].

Four articles using SREP sensitisation as a CS indicator in FM were included in our review [53–56] (Table 1). All of these articles reported greater SREP sensitisation in FM patients compared to HC [53,54,56] and rheumatoid arthritis patients [55,56]. In fact, SREP sensitisation response was only found in FM patients in all of the studies. Regarding secondary findings, SREP sensitisation was positively associated with clinical pain levels [53,56] and other typical FM symptoms [56]. Furthermore, SREP sensitisation was inversely related to blood pressure [54] and proved to be a reliable measure [55].

#### 3.3.9. Evoked pain combined with neuroimaging

Evoked pain measures have also been combined with neuroimaging techniques to assess CS in FM patients [87–89]. Eight such articles were included in our review; four of them used TSP plus functional magnetic resonance imaging (fMRI) [44,52,66,69], while two assessed pressure pain stimuli [70] and maintained cold stimulation [58] in combination with O_2_Hb brain levels using functional near-infrared (fNIR) spectroscopy, one combined secondary hyperalgesia and fMRI assessments [47], and another combined measures of pain perception to pressure stimulation and cerebral blow flow (CBF) using functional transcranial Doppler (fTCD) ultrasonography [62].

Regarding the first of the six articles introduced above, Burgmer *et al.* associated the more severe secondary hyperalgesia seen in FM patients compared to HC with impairment of central pain inhibition, in turn, related to the cerebral mid-brain mechanisms implicated in pain transmission [47]. Meanwhile, Üceyler *et al.* found higher O_2_Hb levels in FM patients than HC in brain areas related to pain processing, such as the dorsolateral prefrontal cortex, in response to repeated painful stimuli [70]. In response to cold pain stimulation, Donadel *et al.* observed a lower change in O_2_Hb concentrations in the left prefrontal cortex of FM patients in comparison with HC, suggesting dysfunction in this inhibitory pain-related brain area [58]. This specific difference allows for discrimination greater than 80% between FM patients and HC [58]. Montoro *et al.* observed a complex pattern of temporal CBF changes in response to pressure pain stimulation, with alterations seen in most stages of the response in FM patients relative to HC; this suggested the presence of CS in FM [62]. Using fMRI combined with a TSP procedure, Bosma *et al.* observed a lower activation in dorsal horn and brain areas related to pain inhibition (the rostral ventromedial medulla and periaqueductal grey region) in FM patients than in HC [44]. Moreover, greater AS was associated with a greater influence of dorsal horn activity on pain [44]. Craggs *et al.* study was the only one that did not report differences between FM and HC in pain-related brain activity in response to a TSP procedure [52]. Schreiber *et al.* combined a TSP procedure with fMRI, which revealed greater brain activity of the medial temporal lobe in FM than in HC; this was also seen in the context of AS [66]. Lastly, Staud *et al.* observed a larger extent of spinal cord involvement and a greater activity of pain-related brain areas during TSP evocation in FM patients in comparison with HC [69]. More details are provided in Table 1.

### 3.4. Risk of bias

The Cochrane ROB evaluation was performed independently by C.M.G.S. and C.I.M., except for articles that they authored; in such cases, P.d.l.C. or G.A.R.d.P carried out the ROB evaluation. Thus, no article was evaluated by an author thereof. The initial agreement rate was 95%. The consensus was achieved either through discussing how the criteria were interpreted again or based on the input of the senior reviewer (G.A.R.d.P). The ROB evaluation revealed that 3 studies were of high quality [42,51,72], 3 were of moderate quality [46,54,59], and the reimaging 28 studies were of low quality. Details on the ROB assessments are shown in Table 2.

Further limitations were identified by the authors. These were the non-indication of the diagnostic criteria [52], possible group small size [42–44,46,50,52–55,59,64,65,69,70,72], failure to specify the method used to determine the sample size [41–43,47,50,52–58,61–63,65,66,69–71,73,74] failure to report any measure of the effect size [41,43–45,47,49–52,57,58,61,64–68,70,71,74], and the non-report of study limitations [41,61,62,65,73]. Besides, some of the selected studies used the same cohort, namely those of Coppieters *et al.* [51] and de la Coba *et al.* [56], which used the cohorts of Coppieters *et al.* [50] and (in part) de la Coba *et al.* [55], respectively.

## 4. Discussion

In the present systematic review, the dynamic evoked pain measures used most frequently during the last few years to effectively assess CS processes in FM were identified. In addition, the future of algometry as a method to assess CS involvement in these patients was analysed. Studies published during the last decade including FM patients and dynamic evoked pain indicators, specifically designed to assess augmented responsiveness of the CNS, were reviewed.

Regarding the dynamic evoked pain measures used by the studies in this review, higher efficacy was seen for the TSP/WU, WU ratio, WU function, AS, and RISP (all of which are derived from or have potential as TSP-related measures) as indicators of CS in FM patients compared to other CS pain measures (e.g. SSP, CPM, and the NFR threshold). In general, the TSP-related pain measures demonstrated greater CS-related pain responses in FM patients compared to HC [44,45,52,59,66,68,69] and other medical conditions [50,53,55]. In this regard, though TSP-related pain measures failed to reveal group differences in CS levels in four studies [43,45,60,69], one of them compared FM with a similar CS condition as chronic fatigue syndrome [45]. Strikingly, associations between most of those indicators (except AS) and the clinical pain levels of FM patients were rarely reported [68]. In spite of this, it is known that TSP has limited power to determine the extent of CS pain involvement in FM on an individual basis, i.e. it is most effective for assessing large samples of patients [90].

In contrast to the TSP-related indicators mentioned above, SSP was less effective for determining the levels of CS to pain in FM patients. Of the two studies using SPP, only one compared FM patients with controls (HC and a low back pain group); it reported no group differences [59]. The other study used SSP to quantify changes in CS levels after a physiologic education intervention in FM patients and demonstrated encouraging outcomes [72]. However, these findings should be treated with caution considering the small number of SSP studies included in the present systematic review.

Regarding CPM and the NFR threshold, although half of the studies reviewed reported greater CS in FM compared to HC and/or patients with other medical conditions [45,46,48,49,57], the other half did not observe group differences in CS levels [50,51,59,64,71,74]. Thus, the CPM and NFR findings may be considered equivocal. As a possible explanation for this, CPM and NFR share a limitation, namely wide variation in the protocols used to evoke NFR and CPM responses [84–91]. In comparison, TSP (based on the WU effect) is more easily evoked using protocols based on repeated stimulation at a frequency ≥ 0.33 Hz [8]. It should also be noted that, in the studies reporting lower CPM in FM patients, this was consistently associated with higher BDNF levels [46,48,67]; this could be interpreted as objective evidence that deficient CPM in FM patients can serve as a CS indicator. In line with this, recovery of CPM after treatment of FM patients has been demonstrated [73], as well as an inverse association between CPM and the severity of FM symptoms [67]. Nonetheless, Bourke *et al.* did not find associations between CPM and clinical pain, physical function, fatigue or anxiety in these patients, though inefficient CPM was related to enhanced TSP [45]. Regarding the NFR threshold, the reported modulatory effect of depressive mood and affective states on this pain indicator is also notable [42,57,64,71]. This confounding effect might preclude exploration of the associations among the NFR threshold, clinical pain [42], and pain sensitivity [49,74] in FM patients.

Despite the limited evidence regarding the efficacy of CuSP as a CS indicator, two studies reported differences between FM patients and HC in CuSP parameters [41,65]. Both studies explored the evocation of the spinal inhibitory response through the latency and duration of CuSP, and both revealed deficiencies in pain inhibition in FM. Given the above findings and scant evidence for the efficacy of CuSP as a CS pain indicator, further research is needed. Future studies should explore the suitability of including CuSP in QST protocols to evaluate CS.

On other hand, SREP has proven to be a dynamic evoked pain technique that is highly sensitive to the augmented responsiveness to pain that characterises FM. Although the underlying mechanisms of SREP remain unknown, its positive association with clinical pain [53], and higher reliability [55] and diagnostic accuracy compared to TSP for discriminating FM patients from HC [53,54,56] and those with other non-CS pain conditions like rheumatoid arthritis [55], support its use in clinical practice. Nevertheless, further research on the physiological basis of SREP is needed.

Finally, the reviewed studies combining algometry with neuroimaging suggest that imaging modalities such as fMRI allow for more objective quantification of CS. In line with this, seven of the eight reviewed studies using evoked pain indicators showed that the application of painful stimulation in FM patients was clearly associated with greater activation of central areas related to pain facilitation, and/or with lower activation of areas related to pain inhibition in comparison to HC [44,47,58,62,66,68,69]. In turn, the studies using dynamic pain indicators sensitive to CS together with fMRI reported a physiological correlation between the increasing pain responses to TSP and AS, manifesting as less activation of brain areas and spinal pathways related to pain inhibition, and greater activation of those related to pain facilitation [44,66,69]. No significant differences in the pain-related brain areas implicated in the TSP response were observed by Craggs *et al.* [52]. All these findings support the combined use of neuroimaging and evoked painful stimulation protocols, especially those based on dynamic evoked pain, to more comprehensively assess CS processes in FM patients. In addition, the high temporal resolution of neuroimaging techniques such as fNIR spectroscopy [58,70] and fTCD ultrasonography [62] allows almost real-time assessment of the CS processes in response to painful stimulation.

Each one of these dynamic evoked pain measures has different benefits and drawbacks, as indicated by the present systematic review. Despite their limitations, to some extent, they all have the ability to discriminate the CS processes involved in the pain pathophysiology of FM patients. Indeed, this review supports the recommendations of using evoked pain measures together with clinical pain reports [92] and tender point count [22] for CS assessments of FM patients. Also, combined use of algometry and simultaneous neuroimaging techniques is recommended to obtain deeper insight into the specific mechanisms underlying CS to pain. However, it must be emphasised that neuroimaging cannot replace pain reports, although it can improve understanding of the neural mechanisms underlying pain experience. Thus, its use is mainly limited to the research setting, also taking into account the high costs associated with applying the technique for screening purposes in clinical settings [93]. Another key recommendation for accurate interpretation of pain responses, based on a large proportion of the reviewed studies, is combining algometry with an assessment of psycho-affective and psychosocial factors known to influence the pain experience, especially: pain catastrophizing, anxiety, and depression.

This systematic review not only revealed the dynamic evoked pain indicators most useful to assess CS in FM but also identified some less well-known ones, such as CuSP, RISP and the recently developed SREP sensitisation. Further development and validation of the specific procedures for CS assessment are necessary to achieve consensus and optimise current QST methods. It is imperative to develop a unified algometry protocol by integrating current QST protocols, some new algometry measures, measurement of the psychological factors affecting pain responsiveness, and perhaps also some relatively accessible neuroimaging techniques to homogenise the assessment of CS processes in FM and other chronic pain conditions.

Some limitations of the present systematic review should be considered. First, we could not control for the diverse pain modalities assessed by the reviewed studies due to insufficient data. Moreover, it should be noted that both skin and muscle (subcutaneous/deep) tissues were differentially stimulated in different body locations using heat, mechanical, and electrical stimulation, with the pain responses obtained suggesting CS involvement in most cases. Thus, these findings suggest that for a more global assessment of CS in FM, the current QST protocols should consider diverse types of stimulation, as well as different sites and tissues for stimulation. Second, the lack of methodological consistency among the studies may account for the differences in findings between the NFR threshold and CPM. Third, other limitations are the own indicated by the authors of reviewed studies (see Risk of Bias section), and the low quality of the majority of these studies in terms of bias assessment (see Table 2). Fourth, despite the well-known sex differences in pain perception in FM [94], all of the reviewed studies included only (or predominantly) female participants. Thus, studies including a greater proportion of males seem to be necessary. Lastly, 5 of the 30 reviewed studies were published by the authors of this review [53–56,62]. However, it is important to note that none of the authors of this review examined the bias quality in their own studies.

In conclusion, this review demonstrated the utility of algometry for examining the involvement of CS processes in FM by itself. Nevertheless, the future of algometry may be dependent on (1) consideration of the psychological factors conditioning the pain experience of FM patients, (2) new dynamic evoked pain indicators, (3) global assessments involving diverse body locations and tissues for stimulation, and (4) the simultaneous use of certain neuroimaging techniques (mainly for research purposes). All this could allow a more optimised analysis of CS-related pain responses using evoked pain protocols from research and clinical practice. However, greater consensus regarding CS measurement methods, and clarification of the underlying mechanisms (e.g. of RISP and SREP sensitisation) are still needed. In conclusion, our findings underline the benefits of algometry for assessing the contribution of CS to FM.

## Data Availability

Data sharing is not applicable to this article as no new data were created or analyzed in this systematic review.

## Professional positions

Pablo de la Coba, PhD, Postdoctoral fellow (https://orcid.org/0000-0001-6469-3553)

Casandra I. Montoro, PhD, Assistant professor of Psychology (https://orcid.org/0000-0003-4145-8600)

Gustavo A. Reyes del Paso, PhD, Professor of Psychology (https://orcid.org/0000-0002-2188-8673)

Carmen M. Galvez-Sánchez, PhD, Postdoctoral fellow (https://orcid.org/0000-0002-1927-0292).
